# Health Care Experiences Among LGBTQ+ Adults: A Qualitative Systematic Review and Meta-Synthesis

**DOI:** 10.1097/jnr.0000000000000750

**Published:** 2026-05-19

**Authors:** Yen-Fan LEE, Te-Sheng CHANG, Wan-Ru WU, An-Chi O-YANG

**Affiliations:** 1 School of Nursing, College of Medicine, National Taiwan University, Taipei, Taiwan; 2 Department of Nursing, College of Nursing, Tzu Chi University, Hualien, Taiwan; 3 Department of Education and Human Potential Development, National Dong Hwa University, Hualien, Taiwan; 4 Department of Post-Baccalaureate Nursing, College of Nursing, Tzu Chi University, Hualien, Taiwan

**Keywords:** LGBTQ+, health care experience, Minority Stress Model, intersectionality, culturally sensitive care

## Abstract

**Background:**

LGBTQ+ (lesbian, gay, bisexual, transgender, and queer) adults often face multilayered discrimination, structural barriers, and cultural exclusion in health care settings that limit their access to care and adversely affect their health outcomes. However, comprehensive syntheses of their health care experiences remain limited.

**Purpose:**

This study was designed to systematically synthesize the health care experiences of LGBTQ+ adults in formal care settings using the Joanna Briggs Institute’s meta-synthesis approach, with a focus on their stressors, care-related challenges, and identity negotiations. These experiences were then interpreted through the lens of the Minority Stress Model to deepen the understanding of health care providers of the primary structural stressors and their implications in health care.

**Methods:**

A qualitative systematic review and meta-aggregation were conducted following the Joanna Briggs Institute (JBI) methodology. Chinese and English databases were searched, and studies were screened, appraised, and synthesized using standard JBI procedures and a ConQual confidence assessment.

**Results:**

Twenty-three qualitative studies were included, yielding 70 findings, 15 categories, and 7 synthesized findings. Key themes identified included heteronormative assumptions, internalized stigma, identity concealment, structural inaccessibility, health care alienation, and care withdrawal. Conversely, affirming care environments and formal/informal social support were identified as enhancing patient trust and identity empowerment.

**Conclusions:**

LGBTQ+ patient experiences reflect structural stress and intersectional inequities in the health care they receive. Structural reforms in clinical practice, health policies, and professional education are needed to promote culturally sensitive, equity-oriented care for LGBTQ+ populations.

## Introduction

LGBTQ+ adults, broadly referring to individuals whose sexual orientation or gender identity falls outside the heterosexual and cisgender majority, encompass diverse sexual and gender identities. The acronym LGBTQ+ includes lesbian, gay, bisexual, transgender, and queer/questioning individuals, while the “+” acknowledges the existence of a broader spectrum of sexual and gender diversity beyond these five categories. Each identity encompassed within this term reflects unique lived experiences, shaped by intersecting aspects of gender, sexuality, and societal expectations (Frost & Meyer, 2023; Logie et al., 2018). In this review, LGBTQ+ adults comprised the focus of analysis, with particular attention given to their marginalized positions within social structures and health care systems.

The World Health Organization affirms that sexual orientation and gender identity must not be grounds for health care discrimination and urges states to implement gender-inclusive and culturally competent care policies (World Health Organization, 2022). However, LGBTQ+ adults often encounter significant barriers within health care systems that contribute to disparities in physical and mental health outcomes. These barriers include societal stigma (Haase et al., 2022; Jackson et al., 2021), discrimination by health care providers (Ayhan et al., 2020; Scott, Gaska, et al., 2023), institutional lack of cultural competence (Uysal-Toraman & Agartioglu-Kundakci, 2018), and policies that fail to address the specific needs of sexual and gender minorities (Smart et al., 2022). Collectively, these challenges can lead to delayed care, avoidance of health services, and insufficient treatment (Graham et al., 2011; Scott, Knopp, et al., 2023; Suen & Chan, 2020). A systematic review further found that 2%–41% of gender-diverse patients reported discrimination in health care settings, which is a factor associated with subsequent help-seeking avoidance (Ayhan et al., 2020). Moreover, outside of clinical settings, LGBTQ+ individuals also face challenges, including societal stigma, institutional lack of cultural competence, and policy-level failures to address their specific needs (Ayhan et al., 2020; Scott, Gaska, et al., 2023). These intersecting barriers contribute to health care avoidance, mistrust, and cumulative health inequities.

Intersectionality theory, initially coined by Kimberlé Crenshaw (Ruiz et al., 2021), highlights how overlapping social identities such as sexual orientation, gender identity, race, class, and immigration background interact to produce unique forms of disadvantage. Individuals with multiple marginalized identities, for example, being a racial as well as gender minority, are more likely to experience institutional exclusion from health services due to medical stigma, language barriers, and limited access to care (Bi et al., 2019; Joudeh et al., 2021). Intersectional theory is a valuable lens for understanding how these structural factors intersect, constraining access to appropriate services and an identity-affirming environment (Lacombe-Duncan et al., 2022). Incorporating this framework into health research facilitates the deeper analysis of within-group differences and promotes the development of more equitable and culturally responsive health care systems.

In addition to direct discrimination, LGBTQ+ adults often face internal stressors such as anticipated stigma, identity concealment, and internalized homophobia or transphobia that contribute to long-term psychological distress and disengagement from health care (Frost & Meyer, 2023). Combined with systemic and institutional barriers, these proximal stressors create a multilevel burden on health. Although cultural shifts such as increased legal protections and growing public awareness have raised expectations for inclusive care, progress remains uneven across regions. The coexistence of progressive policies with persistent stigma or misinformation continues to shape access to equitable health care and health outcomes (Logie et al., 2018; Smart et al., 2022). Understanding how these stressors interact with broader sociocultural forces is essential to interpreting LGBTQ+ health care experiences and highlights the need for a theory-informed synthesis.

LGBTQ+ individuals experience a range of stressors that contribute to health disparities. These include distal stressors, for example, external discrimination, harassment, and structural stigma, and proximal stressors, for example, internalized homophobia or transphobia, identity concealment, and anticipated rejection (Cyrus, 2017). Recent research findings further highlight that individuals with multiple marginalized identities, such as LGBTQ+ people of color, are especially vulnerable to compounded stressors arising from both societal discrimination and exclusion within LGBTQ+ communities. Intracommunity inequities based on race, gender identity, or class may also act as distal stressors and indirectly affect mental health through internalized stress processes (Parmenter & Winter, 2025). These intersecting stressors reflect the cumulative nature of minority stress, which operates across individual, interpersonal, and structural levels to shape health care experiences and long-term outcomes.

Meyer’s Minority Stress Model (MSM) provides a conceptual framework for understanding how LGBTQ+ -specific stressors produce health inequities (Frost & Meyer, 2023; Meyer, 2003). This model distinguishes between distal stressors (e.g., discrimination, violence, and harassment) and proximal stressors (e.g., internalized homophobia or transphobia, concealment of identity, and anticipated rejection), both of which are associated with adverse mental health outcomes (Hendricks & Testa, 2012). Viewed through an ecological lens, these stressors emerge at multiple systemic levels, influencing trust in providers, engagement in care, and overall health outcomes (Moorhead et al., 2024).

The discrimination and structural barriers encountered by LGBTQ+ adults in health care have been examined in several recent qualitative systematic reviews. However, many of these reviews focus on specific health concerns such as mental illness, sexual assault, or HIV-related care, which limits the generalizability of their findings to broader health care experiences (Edwards et al., 2023; Marshall & Cahill, 2022; Rees et al., 2021). In addition, many reviews have centered on specific subpopulations (e.g., transgender individuals) who are disproportionately affected by discrimination (Kcomt, 2019). Few studies have been designed to explore systematically how within-group differences and intersecting identities (e.g., race, age, socioeconomic status) shape health care experiences, highlighting a significant gap in the literature.

In this review, the term “cultural challenges” refers to both institutional and sociocultural norms (e.g., cisnormativity, heteronormativity, medical gatekeeping, and language barriers) that shape how LGBTQ+ adults interact with health care. These cultural factors stem from dominant societal values, clinical practices, and policy frameworks, and influence how LGBTQ+ individuals navigate care systems and negotiate their identity in formal health care settings.

While qualitative research has been employed to elucidate the lived experiences, health care needs, and decision-making processes of LGBTQ+ patients (Renjith et al., 2021), the current literature lacks a comprehensive synthesis that integrates identity-based complexity with structural health care dynamics. To address this gap, a qualitative systematic review guided by the MSM and intersectionality theory was conducted in this study to investigate the health care experiences of LGBTQ+ adults in formal health care settings.

## Methods

### Review Design and Review Questions

The qualitative systematic review and meta-synthesis approach adopted in this study follows the evidence synthesis procedures recommended by the Joanna Briggs Institute (JBI; Aromataris & Munn, 2020) and was designed to systematically synthesize the health care experiences of LGBTQ+ adults in formal care settings, with a focus on related stressors, care-related challenges, and identity negotiations. The results were subsequently interpreted through the lens of the MSM to deepen scholarly understanding of the structural stressors faced by LGBTQ+ patients and the impact these structures have on the health care experience of this community.

To guide the synthesis and interpretation process, this review was framed by two research questions, namely (1) What are the health care experiences of LGBTQ+ adults in formal care settings? and (2) What structural barriers and cultural challenges are revealed in their perspectives?

Although various qualitative designs (e.g., phenomenology, thematic analysis, content analysis) were used in the included studies, the JBI meta-aggregation approach, which allows integration across qualitative traditions, was applied in this study. Furthermore, it was presumed that studies demonstrating methodological rigor and clear, descriptive findings add to the comprehensive nature of the synthesis. Therefore, one phenomenological study (Her, 2016), which met 9 out of 10 items on the JBI Critical Appraisal Checklist and contributed thematically relevant findings addressing the review questions, was included in this review.

### Search Strategy and Data Extraction

#### Search strategy

For this study, two independent reviewers conducted systematic literature searches in both English- and Chinese-language databases. The English databases included Cochrane Library, PsycINFO, CINAHL (via EBSCOhost), and PubMed (MEDLINE), and the Chinese databases included the CEPS (Chinese Electronic Periodical Services; Airiti Library) and the National Digital Library of Theses and Dissertations in Taiwan (NDLTD). Search terms used for the databases were: (LGBT OR sexual and gender minority) AND (healthcare OR care) AND (healthcare experience OR experience). The search strategy combined terms related to LGBTQ+ populations, health care, and qualitative research.

While “structural barriers” and “cultural challenges” were not explicitly listed as search keywords, studies were assessed for conceptual relevance to these themes during the screening process. Specifically, we considered whether the initially identified studies addressed discrimination, stigma, health care exclusion, medical mistrust, or other institutional and sociocultural barriers—even if not directly named.

**Figure 1:**
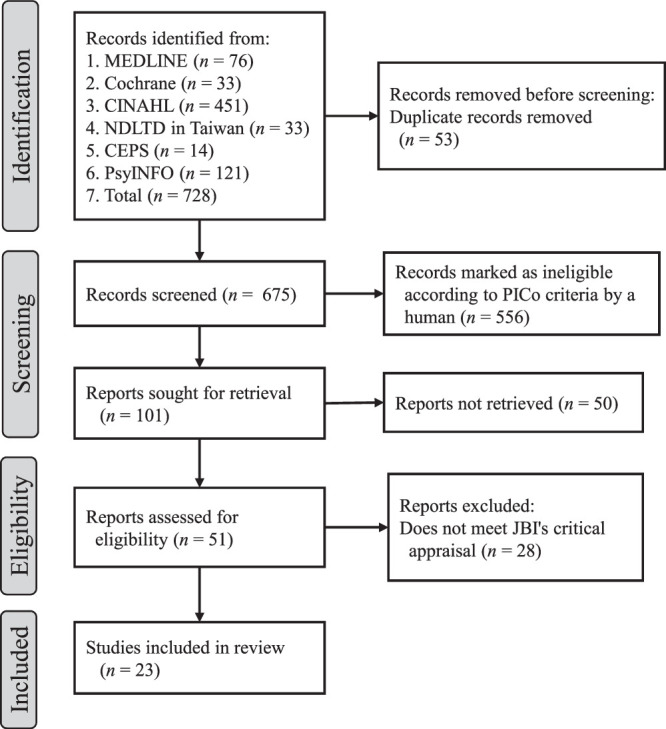
PRISMA 2020 Flow Diagram for Systematic Reviews (Database and Register Search Only) Note. NDLTD = Networked Digital Library of Theses and Dissertations; CEPS = Chinese Electronic Periodical Services; PICo = Population, Phenomenon of Interest, Context; JBI = Joanna Briggs Institute.

In addition, Boolean operators were used to refine the search process. The search covered all of the studies published in each database from its inception to April 2025. The inception years of the databases were PubMed (1996–), CINAHL (1984–), PsycINFO (1967–), Cochrane Library (1993–), CEPS (2002–), and NDLTD (1997–). All of the retrieved articles were imported into EndNote 20 for deduplication and were screened according to the Preferred Reporting Items for Systematic Reviews and Meta-Analyses (PRISMA) 2020 flow diagram (Page et al., 2021). The selection process is illustrated in Figure 1.

#### Inclusion and exclusion criteria

The inclusion criteria for studies were defined using the PICo (Population, Phenomenon of Interest, Context) framework, as follows: (1) reporting on the health care experiences of LGBTQ+ adults (aged 18 and over); (2) focused on health care experiences or interactions; (3) conducted in formal health care settings or involving health care health care providers; (4) using a qualitative approach and approaches such as phenomenology, interpretive study, grounded theory, ethnography, descriptive qualitative research, or the qualitative component of mixed methods research; and (5) published in either English or Chinese. Studies were excluded if they were duplicate publications, lacked a full text, were purely quantitative, or were review or opinion articles.

### Study Selection

Two reviewers independently screened the titles, abstracts, and full texts based on the inclusion and exclusion criteria. Discrepancies were resolved through discussion, and when necessary, a third reviewer with expertise in qualitative research was consulted. The screening process was documented using the PRISMA flow diagram.

Two of the included studies employed less common but valid analytic approaches. One of these, Nadarzynski et al. (2017), used framework analysis, a matrix-based method often used in applied health and policy research that allows data to be compared across cases and themes. The other, Paine (2018), used retrospective analysis, which draws on participants’ reflections of past events and is appropriate for exploring identity or health care trajectories. Both approaches produced thematically organized, participant-derived findings compatible with JBI’s meta-aggregation synthesis method.

### Critical Appraisal

In this review, the JBI Critical Appraisal Checklist for Qualitative Research followed the methodological guidelines of Aromataris and Munn (2020), a mature and valid tool for assessing the quality of qualitative studies in systematic reviews. The JBI checklist comprised 10 criteria: (1) congruity between the research methodology and the stated philosophical perspective, (2) alignment between the methodology and the research question, (3) appropriateness of data collection methods, (4) rigor in data analysis, (5) adequate representation of the findings with supporting evidence, (6) clarity in the researcher’s cultural or theoretical positioning, (7) examination of the researcher’s influence on the research, (8) adherence to ethical principles, (9) approval by an ethics review board, and (10) logical coherence between analysis and conclusions.

The evaluation was conducted independently by two reviewers, both of whom had training and experience in qualitative meta-aggregation and one of whom self-identified as LGBTQ+. Studies that met at least 8 of the 10 abovementioned criteria with a “Yes” rating were deemed methodologically robust and included in the synthesis. Discrepancies in appraisal were first resolved through discussion between the reviewers, with unresolved cases adjudicated by a third reviewer with expertise in qualitative methodology. This rigorous process ensured the reliability and methodological integrity of the included evidence, as well as aligns with JBI’s standards for qualitative systematic reviews.

### Data Extraction

Following the JBI methodology for qualitative systematic reviews, data extraction and synthesis were conducted in an integrated and iterative manner. The extracted information included both study-level characteristics (such as author, year, country, participant demographics, objectives, and methods) and interpretive content (specifically reported findings and supporting participant quotations). All of the data were organized into a standardized extraction matrix.

### Data Synthesis and Integration

A meta-aggregation approach was used to synthesize findings using a structured three-stage process of (1) extracting individual findings, (2) categorizing these into thematic groups, and (3) synthesizing overarching analytical themes. Reflexivity was maintained using independent coding and team consensus discussions throughout this process. To enhance theoretical coherence, the review team revisited the synthesized findings in light of the MSM and intersectionality theory. Rather than applying predetermined classifications, the team examined how the emergent themes resonated with theoretical constructs such as different types of social stressors and resilience processes, thereby grounding the interpretation in both empirical data and established conceptual frameworks.

The synthesis followed the three stages of JBI meta-aggregation. These were (1) Extracting findings: Two reviewers independently extracted individual findings and supporting quotations from the included studies. (2) Categorizing findings: Similar findings were grouped into analytical categories based on shared descriptive meanings. (3) Synthesizing categories: Consensus meetings aggregated categories into synthesized findings reflecting broader themes. This process was iterative and reflexive, with theoretical guidance from the Minority Stress Model and intersectionality used to ensure conceptual coherence (Frost & Meyer, 2023).

### Confidence in Qualitative Findings (ConQual Assessment)

In this review, the ConQual approach (Confidence in the Output of Qualitative Research Synthesis) developed by the Joanna Briggs Institute was followed in assessing the level of confidence associated with each synthesized finding. ConQual is used to evaluate findings based on the two key components of dependability and credibility, with the final level of confidence categorized as high, moderate, low, or very low after applying necessary downgrades.

Dependability was assessed using the five key items from the JBI Critical Appraisal Checklist for Qualitative Research: congruity between the research methodology and, respectively, the research question (item 2), data collection methods (item 3), and data analysis (item 4); acknowledgment of the researcher’s theoretical or cultural position (item 6); and consideration of the influence of the researcher on the research (item 7). Studies that met 4–5 criteria, 2–3 criteria, and 0-1 criteria were respectively considered to have high, moderate, and low levels of dependability (Munn et al., 2014). Moreover, when a majority of studies contributing to a synthesized finding were moderate or low in dependability, overall confidence was downgraded by one level.

Credibility was assessed by evaluating the degree of support given to each study finding by direct quotations, observational data, or other participant-derived evidence. Findings were classified as unequivocal (U), credible (C), or not supported (NS). Synthesized findings that included C- or NS-rated data were downgraded in terms of level of confidence using the following formula: reduction of one level for the inclusion of some C-rated findings, two levels if all findings are C-rated, three levels for the inclusion of some NS-rated findings, and four levels if all findings are NS-rated.

Overall, the ConQual process provides a systematic and transparent methodology for evaluating confidence in qualitative evidence synthesis, yielding a final confidence rating for each synthesized finding.

## Results

The results of the critical appraisal are detailed in Table 1; the data extracted from the included studies are summarized in Appendix, Supplemental Digital Content 1, http://links.lww.com/JNR/A8, and the synthesis results, comprising 70 individual findings, 15 categories, and 7 synthesized findings derived from 23 qualitative studies, are shown in Table 2.

**Table 1:** JBI Critical Appraisal Checklist for Qualitative Research Results for the Included Studies

First Author (Year)	Q1	Q2	Q3	Q4	Q5	Q6	Q7	Q8	Q9	Q10	Score ^a^
Bi (2019)	U	Y	Y	Y	Y	U	Y	Y	Y	Y	8
Bradford (2016)	U	Y	Y	Y	Y	U	Y	Y	Y	Y	8
Bristowe (2018)	U	Y	Y	Y	Y	U	Y	Y	Y	Y	8
Burton (2020)	U	Y	Y	Y	Y	Y	Y	Y	Y	Y	9
Czimbalmos (2022)	U	Y	Y	Y	Y	Y	Y	Y	Y	Y	9
Greene (2019)	Y	Y	Y	Y	Y	Y	Y	Y	Y	Y	10
Hascher (2024)	U	Y	Y	Y	Y	Y	Y	Y	Y	Y	8
Her (2016)	Y	Y	Y	Y	Y	Y	Y	Y	U	Y	9
Higgins (2019)	Y	Y	Y	Y	Y	U	Y	Y	U	Y	8
Holland (2021)	Y	Y	Y	Y	Y	Y	Y	Y	U	Y	9
Hoyt (2020)	Y	Y	Y	Y	Y	Y	Y	Y	Y	Y	10
Joudeh (2021)	Y	Y	Y	Y	Y	Y	Y	Y	Y	Y	10
Kelsall-Knight (2020)	Y	Y	Y	Y	Y	Y	Y	Y	U	Y	9
Kielhold (2024)	U	Y	Y	Y	Y	U	Y	Y	Y	Y	8
Kulshreshtha (2022)	Y	Y	Y	Y	Y	Y	Y	Y	Y	Y	10
Legere (2016)	Y	Y	Y	Y	Y	U	Y	Y	Y	Y	9
Logie (2018)	Y	Y	Y	Y	Y	U	Y	Y	Y	Y	9
Müller (2017)	U	Y	Y	Y	Y	Y	Y	Y	Y	Y	9
Nadarzynski (2017)	U	Y	Y	Y	Y	Y	Y	Y	Y	Y	9
Paine (2018)	Y	Y	Y	Y	Y	Y	Y	Y	Y	Y	10
Rana (2022)	Y	Y	Y	Y	Y	U	Y	Y	Y	Y	9
Smart (2022)	U	Y	Y	Y	Y	U	Y	Y	Y	Y	8
Soinio (2020)	U	Y	Y	Y	Y	U	Y	Y	Y	Y	8

*Note.* Y = yes; U = unclear. Q1: Is there congruity between the philosophical or theoretical positioning of the study and the methodological approach that has been adopted? Q2: Is there congruity between the research methodology and the research question or objectives? Q3: Is there congruity between the research methodology and the methods used to collect data? Q4: Is there congruity between the research methodology and the representation and analysis of data? Q5: Is there congruity between the research methodology and the interpretation of results? Q6: Is there a statement locating the researcher culturally or theoretically? Q7: Is there a statement locating the researcher culturally or theoretically? Q8: Are participants and, where appropriate, their voices adequately represented? Q9: Is the research ethical according to current criteria, and for recent studies, is there evidence of ethical approval by an appropriate body? Q10: Do the conclusions drawn in the research report flow from the analysis or interpretation of the data?

^a^ The total score is 10.

**Table 2:** Findings, Categories, and Synthesized Findings

Finding ^a^	Category	Synthesized Finding
• Discrimination against LGBTQ individuals based on race/ethnicity (Her, 2016; Hoyt, 2020)	Discrimination at the intersection of sexual orientation and ethnocultural identity	Marginalization at identity intersections
• Discrimination against LGBTQ immigrants (Bi, 2019; Hoyt, 2020; Smart, 2022)		
• Discrimination against LGBTQ foreign nationals (Czimbalmos, 2022)		
• Discrimination against LGBTQ individuals living in rural areas (Joudeh, 2021)		
• Discrimination against LGBTQ older adults (Bradford, 2016; Burton, 2020)	Embodied discrimination related to sexual orientation and age/gender	
• Discrimination against LGBTQ women (Greene, 2019; Higgins, 2019; Soinio, 2020)		
• Discrimination against men who have sex with men (Nadarzynski, 2017; Rana, 2022)		
• Discrimination against LGBTQ individuals requiring end-of-life care (Bristowe, 2018)	Care disparities at the intersection of sexual orientation and health status	
• Discrimination against LGBTQ individuals living with HIV (Hoyt, 2020; Kielhold, 2024; Logie, 2018; Müller, 2017)		
• Discrimination against gay men with prostate cancer (Logie, 2018)		
• Discrimination against lesbian and bisexual women with reproductive cancers (Legere, 2016)		
• Heterosexist assumptions by health care providers (Bristowe, 2018; Greene, 2019; Hoyt, 2020; Soinio, 2020)	Heterosexism and racism in health care	Systemic barriers to access
• Heterosexism among health care professionals (Kulshreshtha, 2022; Legere, 2016)		
• Racism in clinical care (Joudeh, 2021)		
• Over-pathologization of LGBTQ+ identities (Bi, 2019)		
• Homophobia, transphobia, and biphobia from providers (Bristowe, 2018; Hascher, 2024; Higgins, 2019; Holland, 2021; Hoyt, 2020; Joudeh, 2021; Legere, 2016)		
• Exclusion of nonbiological lesbian parents (Kelsall-Knight, 2020)		
• Lack of LGBTQ+ medical knowledge among providers (Hascher, 2024; Müller, 2017)	Provider incompetence or training gaps	
• Inadequate care standards for LGBTQ+ populations (Bristowe, 2018; Soinio, 2020)		
• Inability to support LGBTQ+ sexual assault survivors (Holland, 2021)		
• Negative provider attitudes (Bristowe, 2018; Hascher, 2024; Holland, 2021; Hoyt, 2020; Kelsall-Knight, 2020; Kulshreshtha, 2022; Paine, 2018)		
• Lack of LGBTQ-inclusive health policies (Czimbalmos, 2022; Smart, 2022)	Health system structural inequities	
• Legal system inequities (Bristowe, 2018; Logie, 2018)		
• Barriers to care access (Müller, 2017)		
• Inadequate access to public health information (Müller, 2017; Nadarzynski, 2017)		
• Unequal distribution of health care resources (Czimbalmos, 2022; Hoyt, 2020; Joudeh, 2021; Logie, 2018)		
• Concealing sexual orientation and posing as heterosexual (Bradford, 2016; Burton, 2020; Higgins, 2019; Legere, 2016; Paine, 2018)	Sexual identity dilemma	Disengagement driven by stigma
• Downplaying identity and focusing solely on medical issues (Bi, 2019; Bristowe, 2018)		
• Feeling embarrassed and anxious during the coming-out process (Greene, 2019; Soinio, 2020)		
• Internalized homophobia (Hoyt, 2020; Kulshreshtha, 2022)		
• Internalized stigma (Her, 2016; Hoyt, 2020; Logie, 2018)		
• Anticipated discrimination or stigma in health care interactions (Greene, 2019; Higgins, 2019; Holland, 2021; Müller, 2017)	Health care mistrust	
• Distrust or concern regarding the medical process (Bristowe, 2018; Higgins, 2019; Holland, 2021; Joudeh, 2021; Kielhold, 2024; Paine, 2018; Soinio, 2020)		
• Inadequate access to health care resources (Bradford, 2016; Müller, 2017)	Structural accessibility stressors	Structural and contextual stressors
• Health care providers are overburdened, leading to long wait times (Hascher, 2024; Müller, 2017; Rana, 2022)		
• Institutional complexity and administrative barriers (Rana, 2022)		
• General life-stage stressors (Bristowe, 2018)	Contextual vulnerability stressors	
• Rejection or alienation from family of origin (Bi, 2019; Bristowe, 2018; Hoyt, 2020; Smart, 2022)		
• Economic vulnerability (Bradford, 2016; Czimbalmos, 2022; Müller, 2017)		
• Physical frailty (Bradford, 2016; Burton, 2020)		
• Language barriers (Czimbalmos, 2022)		
• Religious bias (Bi, 2019; Burton, 2020; Her, 2016; Joudeh, 2021; Müller, 2017)		
• Cultural constraints related to ethnic minority status (Bi, 2019; Her, 2016; Higgins, 2019; Kulshreshtha, 2022)		
• Defensive responses to discriminatory behaviors (Joudeh, 2021; Paine, 2018)	Care withdrawal and interactional disengagement in health care	Care avoidance as a coping strategy
• Changing health care providers or institutions (Kielhold, 2024; Müller, 2017)		
• Delaying or avoiding continuous care, resulting in treatment interruption (Kielhold, 2024; Müller, 2017; Paine, 2018; Smart, 2022)		
• Being excluded from the protective umbrella of health care (Higgins, 2019; Holland, 2021; Nadarzynski, 2017)		
• Being marginalized or overlooked during health care interactions (Kelsall-Knight, 2020; Müller, 2017)		
• Contraceptive education for queer women is often overlooked (Higgins, 2019)	Lack of adequate mental, reproductive, and sexual health resources leads to unmet needs among LGBTQ+ individuals	Cumulative risks from service gaps
• Inability to access therapists for mental health concerns (Her, 2016)		
• Prostate cancer receives passive or inadequate treatment (Hoyt, 2020)		
• Lack of access to HIV prevention resources (Logie, 2018)		
• Inadequate access to appropriate sexual and reproductive health care (Greene, 2019)		
• Providers’ cultural competence and continuing education (Bradford, 2016; Greene, 2019; Hascher, 2024; Her, 2016; Kielhold, 2024; Müller, 2017; Soinio, 2020)	Affirming care environment	Trust and empowerment in affirming care
• Active and friendly provider-patient interactions (Bristowe, 2018; Burton, 2020; Holland, 2021; Hoyt, 2020; Kulshreshtha, 2022)		
• Patient-centered and individualized care (Hascher, 2024)		
• LGBTQ-friendly signage and waiting room atmosphere (Greene, 2019; Rana, 2022)		
• Support from family of origin (Hoyt, 2020; Legere, 2016)	Formal/informal social support	
• Support from friends or peers (Hoyt, 2020)		
• Support from a partner (Legere, 2016)		
• Community or social network support (Bristowe, 2018; Logie, 2018)		
• Support from LGBT providers and organizations (Bi, 2019)		
• Support from health care institutions (Bristowe, 2018)		
• Advocacy and legal support through health policy (Hascher, 2024; Kulshreshtha, 2022; Smart, 2022; Soinio, 2020)		
• Seeking health care providers of the same sexual orientation or ethnicity (Bi, 2019)	Identity empowerment	
• Visiting only LGBTQ-affirming health care providers (Hascher, 2024)		
• Having privileged or advantageous social positions (Czimbalmos, 2022)		
• Active engagement in health and care decision-making (Hascher, 2024)		
• The coming-out process contributes to identity empowerment in health care contexts (Higgins, 2019; Kelsall-Knight, 2020; Nadarzynski, 2017)		

^a^ For cited references, only the first author and the publication year are provided.

**Figure 2:**
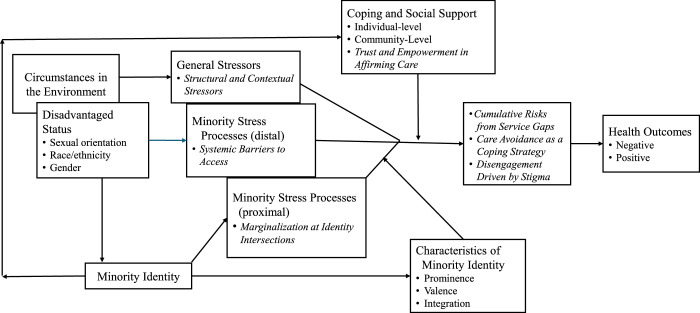
Adapted and Expanded Minority Stress Model (MSM): Intersectionality Lens-Informed Framework Highlighting How Overlapping Marginalized Identities Shape Stress and Health Outcomes Note. Italicized words indicates the seven synthesized findings derived from the systematic literature review.

To illustrate how these synthesized findings were theoretically organized, an adapted version of Meyer’s MSM was developed (Figure 2). This expanded framework integrates key themes such as intersectional marginalization, structural barriers, and coping dynamics into the original MSM to better reflect the health care experiences of LGBTQ+ individuals.

### Finding 1: Marginalization at Identity Intersections

Intersecting identities (e.g., gender, race, and sexual orientation) were shown to intensify internalized stigma and expectations of rejection, reflecting the proximal stress processes described in the MSM framework.

In health care settings, LGBTQ+ adults experience marginalization at the intersection of sexual orientation, ethnicity, age, gender, and health status. Those from immigrant families or conservative religious regions often encounter compounded exclusion and mistrust within the system. One participant shared, “We’re in the South—what they call the Bible Belt. There’s always conflict when people help with hormone therapy” (Joudeh et al., 2021). Others noted being overlooked due to both sexual orientation and gender identity, for example, “You’re gay, and then they completely ignore (contraception). So in my experience, it seems pretty black and white” (Greene et al., 2019). Older adults and women who are lesbian or bisexual reported frequent invisibility and unmet needs (Bradford et al., 2016; Greene et al., 2019). Those living with HIV or chronic conditions reported facing additional barriers to sustained care and equitable access (Bristowe et al., 2018; Logie et al., 2018). The additional difficulties associated with layered disadvantages reveal the limits of single-axis approaches and call for intersectionally responsive care frameworks that recognize the diverse realities faced by LGBTQ+ patients.

### Finding 2: Systemic Barriers to Access

Health care exclusion through discriminatory policies and provider biases was found to manifest as external prejudice events that are consistent with distal stressors in the MSM.

LGBTQ+ adults often face systemic oppression in health care associated with heteronormative assumptions, religious and racial prejudice, and a lack of cultural competence. As one participant explained, “Available therapists are either passively or actively homophobic or racist” (Joudeh et al., 2021). In clinical encounters, LGBTQ+ adults are frequently misidentified or dismissed. “Even after being told twenty times that I was his partner, the consultant still referred to me as his brother” (Bristowe et al., 2018). Moreover, assumptions such as the belief that lesbian women are not at risk for HIV, leading to a denial of testing services, undermine care quality (Müller, 2017). These structural prejudices are further compounded by the absence of inclusive health policies and unequal resource distribution, resulting in diminished trust and access that reinforces long-standing disparities in care for LGBTQ+ populations.

### Finding 3: Disengagement Driven by Stigma

Health care exclusion through discriminatory policies and provider biases was found to manifest as external prejudice events, which is consistent with distal stressors in the MSM.

This finding highlights how pressures surrounding sexual identity and internalized stigma can lead to withdrawal and disengagement among LGBTQ+ adults during health care encounters. To avoid potential discrimination, many conceal their identities or discuss illness-specific issues only. As one participant shared, “I lied to get what I needed, then left” (Greene et al., 2019). These defensive interactions often reflect underlying shame and anxiety, which erode LGBTQ+ adults’ trust in health care providers. When clinicians lack affirming language and cultural awareness, patients struggle to feel supported (Holland et al., 2021). This stigma-driven disengagement compromises care continuity and leaves health needs unmet, contributing to long-term psychological and physical health disparities.

### Finding 4: Structural and Contextual Stressors

Beyond identity-specific discrimination, the participants in the included studies reported experiencing institutional neglect and normative pressures, which functioned as general stressors shaping their health care experiences.

This finding reveals LGBTQ+ adults face compounded barriers not only from sexual orientation–related discrimination but also from structural stressors and contextual vulnerabilities. Barriers such as limited resource availability, overburdened staff, complex administrative processes, and poor transportation access further restrict care engagement (Bradford et al., 2016; Rana et al., 2022). In addition, religious doctrine, cultural discord, and lack of familial support intensify the perceived level of exclusion. As one respondent shared, “Many Christian health care practices bring in religious doctrine, which creates awkwardness or a sense of rupture” (Joudeh et al., 2021). These generally non–sexuality-specific burdens, when layered with LGBTQ+ identities, heighten marginalization and underscore the urgency for equitable, culturally responsive health care systems.

### Finding 5: Care Avoidance as a Coping Strategy

Avoiding health care services was described in these studies as a self-preserving behavior pursued in response to anticipated stigma, suggesting its role as a coping-based mediator within the stress–health link.

This finding reveals LGBTQ+ patients often resort to defensive strategies such as delaying care, switching health care providers, and discontinuing treatment when facing discrimination, exclusion, or neglect within health care systems (Kielhold et al., 2024; Müller, 2017; Paine, 2018). These actions signify a breakdown of trust and lead to disrupted continuity of care, increasing long-term health risks (Smart et al., 2022). Another lesbian parent remarked, “They wrote I was the father (on the medical record) … I mean, how ridiculous is that?” (Kelsall-Knight & Sudron, 2020). Such experiences highlight how misrecognition and cumulative exclusion drive disengagement from care. Clinical practice must address these patterns by fostering culturally sensitive and identity-affirming environments to restore trust and strengthen ongoing patient-health care provider relationships.

### Finding 6: Cumulative Risks from Service Gaps

The compounding effects of delayed, fragmented, and inaccessible care were found to mediate the relationship between systemic exclusion and deteriorating mental health.

Persistent service gaps in LGBTQ+ care, particularly in the realms of mental, sexual, and reproductive health, were identified in this review. For example, heteronormative assumptions were often associated with the exclusion of queer women from contraceptive education, with one patient recalling being asked, “Why aren’t you using contraception?” despite her clearly stating she was in a same-sex relationship (Higgins et al., 2019). Also, LGBTQ+ partners are frequently excluded from medical discussions, which leaves them feeling unwelcome and unsupported (Hoyt et al., 2020). These gaps result in prolonged unmet needs and accumulating health risks, especially in patients dealing with mental health issues such as depression, anxiety, and trauma. Health care systems must address these structural deficiencies by adopting and using inclusive and culturally competent models of care.

### Finding 7: Trust and Empowerment in Affirming Care

Trust-building and identity affirmation in inclusive settings were found to function as protective social resources, spanning both individual and community-level support mechanisms within the MSM.

This finding highlights that culturally sensitive and affirming health care environments play a significant role in fostering trust and engagement among LGBTQ+ patients (Bradford et al., 2016; Greene et al., 2019). Inclusive signage, welcoming spaces, and respectful communication contribute to a sense of safety and validation in clinical encounters (Bristowe et al., 2018). As one participant shared, “Having a ‘gay-friendly’ doctor makes us feel more comfortable” (Hoyt et al., 2020). Empowerment also arises when individuals can choose LGBTQ+-affirming health care providers or gain pride and autonomy in their coming-out process. One respondent reflected, “Once I found feminism and pride in my queer identity, I decided, ‘I want to take control of my body and contraception’” (Higgins et al., 2019). These affirming practices, coupled with broader social support and advocacy, play a critical role in reducing health care isolation and enhancing engagement.

### ConQual Summary of Synthesized Findings

Following the JBI ConQual approach, five of the seven synthesized findings were rated with a high confidence level, while the remaining two were rated at a moderate level of confidence. The ConQual Summary of Findings, presented in Table 3, includes the confidence ratings and reasons for any downgrading of scores. All 23 of the included studies demonstrated high dependability, and most met the criteria for unequivocal credibility. Only three findings from Bradford et al. (2016) were rated as “credible” due to a limited number of directly supporting quotations. Overall, the synthesized findings of this review, underpinned by strong methodological rigor and trustworthy qualitative evidence, offer reliable insights into the health care experiences of LGBTQ+ populations.

**Table 3:** ConQual Summary of Synthesized Findings

Synthesized Finding	Type of Research	Dependability	Credibility	Overall ConQual Score
1. Marginalization at identity intersections	Qualitative	High	C	Moderate
2. Systemic barriers to access	Qualitative	High	U	High
3. Disengagement driven by stigma	Qualitative	High	U	High
4. Structural and contextual stressors	Qualitative	High	U	High
5. Care avoidance as a coping strategy	Qualitative	High	U	High
6. Cumulative risks from service gaps	Qualitative	High	U	High
7. Trust and empowerment in affirming care	Qualitative	High	C	Moderate

*Note.* C = credible; U = unequivocal.

## Discussion

The qualitative findings from 23 studies synthesized in this review reveal how LGBTQ+ adults experience health care through the lens of intersectionality and the MSM. The findings highlight both the barriers and the strengths present in formal care systems, offering a theory-informed understanding of how structural, interpersonal, and intrapersonal factors shape health outcomes.

Several of the synthesized findings identified affirming care environments as protective spaces for LGBTQ+ patients. Affirming care environments are marked by visible indicators of inclusivity (e.g., rainbow signage), gender-neutral forms and language, and respectful use of names and pronouns aligned with patients’ identities (Bristowe et al., 2018; Greene et al., 2019). The participants noted that health care providers who conveyed understanding and acceptance made them feel safer and more willing to engage in open communication (Hoyt et al., 2020). Within the MSM, affirming environments act as protective factors that buffer the impact of distal stressors such as structural stigma and may ameliorate/reduce the severity of proximal stress responses such as identity concealment and hypervigilance (Frost & Meyer, 2023).

The findings of this review also highlight the role of identity empowerment as a key enabler of patient agency among LGBTQ+ adults. Participants who were able to come out in safe clinical environments, choose LGBTQ+-friendly health care providers, and/or witness recognition of their identities generally expressed greater willingness to participate in health care decisions and maintain continuity of care (Hascher et al., 2024; Higgins et al., 2019). These experiences reflected not only enhanced self-acceptance but also a strengthened sense of control in clinical encounters. Within the MSM, identity empowerment may be interpreted as an active coping strategy that counters the effects of internalized stigma and facilitates health-promoting behaviors such as seeking care and sustaining relationships with health care providers (Hoyt et al., 2020).

Many of the LGBTQ+ participants in the included studies self-reported experiencing internalized stigma, which is a form of self-directed shame stemming from long-standing exposure to societal heteronormativity and discrimination. This psychological burden diminishes motivation to seek care and often leads to emotional detachment from health care settings (Her, 2016; Logie et al., 2018). Some further expressed the belief that their sexual or gender identity made them unworthy of empathy or competent care (Hoyt et al., 2020). Within the MSM, internalized stigma is categorized as a proximal stressor that directly undermines self-worth and health behaviors (Frost & Meyer, 2023). Internalized stigma has been positively associated in the literature with anxiety, depression, and delayed health care engagement. In unsupportive health care contexts, these effects are exacerbated, reinforcing avoidance and self-silencing.

Many LGBTQ+ patients resort to identity concealment as a defensive strategy to avoid anticipated discrimination during medical encounters. This includes withholding information regarding sexual orientation, relationships, and sexual health behaviors (Bradford et al., 2016; Greene et al., 2019). While potentially offering short-term protection, concealment compromises health care provider-patient communications and may result in misdiagnoses or suboptimal care (Paine, 2018). Under the MSM, identity concealment is a proximal stress response driven by the expectation of stigma (Frost & Meyer, 2023). Patients often “pass” as heterosexual or remain silent if anticipating negative reactions from health care providers (Legere & MacDonnell, 2016). This unequal dynamic restricts disclosure and reinforces disengagement, ultimately undermining both trust and clinical effectiveness.

Also, the findings of this review indicate LGBTQ+ adults often face structural accessibility barriers attributable to institutional procedures, spatial arrangements, and under-resourced care systems. Long wait times, administrative complexity, and service designs that fail to account for gender diversity contribute to an environment of exclusion (Müller, 2017; Rana et al., 2022). For LGBTQ+ adults with intersecting marginalized identities (e.g., ethnic minorities or migrants), language barriers, religious-affiliated health systems, and culturally insensitive protocols often further intensify structural exclusion (Bi et al., 2019; Czimbalmos & Rask, 2022). Within the MSM, these barriers represent distal stressors that operate not through individual interactions but through broader institutional inequalities in policies, norms, and resource distribution (Frost & Meyer, 2023). These inaccessible environments contribute to cumulative disadvantages that further exacerbate the health vulnerability of LGBTQ+ adults.

In response to structural discrimination and anticipated stigma, LGBTQ+ patients often adopt disengagement strategies to protect themselves. These behaviors include switching health care providers, delaying follow-up care, or fully withdrawing from the health care system (Kielhold et al., 2024; Paine, 2018). While potentially offering temporary psychological relief, these strategies carry increased risks of delayed diagnoses, deteriorating health, and poor mental health outcomes (Smart et al., 2022). Under the MSM, this withdrawal behavior represents a secondary coping mechanism triggered by sustained exposure to distal stressors in the absence of adequate institutional support (Frost & Meyer, 2023). Over time, these avoidance behaviors disrupt chronic care management and reinforce inequities in terms of both access and outcomes.

The synthesized findings of this review demonstrate that formal and informal sources of social support play critical moderating roles in shaping the health care experiences of LGBTQ+ patients. In the included studies, support from partners, family, friends, LGBTQ+ communities, and affirming care organizations were shown to buffer the adverse effects of discrimination and marginalization (Bristowe et al., 2018; Logie et al., 2018), with participants who had access to supportive networks more likely to disclose their identities, articulate their needs, and actively engage in health-related decision-making (Bi et al., 2019; Legere & MacDonnell, 2016). Within the MSM, social support functions as a key moderator that weakens the impact of both distal stressors (e.g., societal stigma) and proximal stressors (e.g., internalized homonegativity) on health outcomes (Frost & Meyer, 2023). When support sources such as LGBTQ+-affirming health care providers or peer-led support groups integrate both identity validation and health empowerment, they foster positive coping and enhance health care engagement and trust.

In light of the seven synthesized findings of this review, the experiences of LGBTQ+ adults in formal health care settings reflect multiple layers of structural, interpersonal, and internalized stress. These findings map closely onto the MSM, including distal stressors (e.g., discrimination, institutional exclusion), proximal stressors (e.g., internalized stigma, identity concealment), and protective factors (e.g., affirming care, social support). Moreover, the incorporation of intersectionality theory addressed the limitations of MSM by accounting for the compounded effects of health care marginalization of race, gender, class, and migration status. Together, these theoretical frameworks offer a more comprehensive lens through which to understand the health disparities faced by LGBTQ+ patients and to support the development of theory-driven interventions in clinical practice, policy, and future research.

### Limitations

Even with the effort invested by the authors to ensure the rigor of this qualitative meta-synthesis, several limitations must be acknowledged. First, this review only included studies published in either English or Chinese. Thus, culturally relevant insights from non-English-speaking and non-Chinese-speaking regions may have been excluded. Given the diversity in health care systems and cultural norms about sexuality and gender around the world, this language restriction may have biased the findings toward Western-centric perspectives.

In addition, 13 of the 23 included studies were conducted in the United States, where the legal status of LGBTQ+ rights, health care provider training, and institutional policies may differ substantially from other regions. These sociocultural conditions may shape how LGBTQ+ individuals perceive stigma, seek support, and navigate medical systems. While the focus on U.S.-based studies offers insight into one particular context, it may limit the generalizability of our findings to non-Western or resource-limited settings, where different cultural, legal, and religious frameworks influence both health care access and LGBTQ+ rights. Future research should increase the geographic diversity covered to capture LGBTQ+ health care experiences across various cultural settings.

Second, most of the included studies focused on high-income countries, primarily in North America and Europe. This geographic concentration limits the transferability of the findings to low- and middle-income countries, where health care infrastructures and LGBTQ+ rights differ significantly.

Third, the MSM was used to guide the synthesis in this review study, although many of the primary studies did not, requiring interpretive mapping to be used across the included studies. Thus, certain findings were conceptually mapped onto the model retrospectively, rather than originally framed by the MSM in the primary studies. Moreover, heterogeneities among participant identities spanning sexual orientations, gender identities, racial/ethnic groups, and health conditions complicate fine-grained analysis efforts. While enriching the synthesis, this diversity necessitates caution when drawing generalizations.

Finally, qualitative data inherently reflect the subjectivity of both participants and researchers. Although efforts were made to preserve the integrity of the findings of each study, the process of thematic synthesis unavoidably involves a degree of abstraction and reinterpretation. To address this potential bias, future meta-syntheses may employ methodological triangulation or participant validation to further strengthen interpretive credibility.

### Implications for Practice, Research, and Policy

The findings of this review have important implications across clinical, research, and policy domains.

Health care institutions should proactively build environments that are culturally sensitive and LGBTQ+-affirming. Key strategies to achieve this include using gender-neutral language, appropriately revising clinical forms and electronic health record systems, and implementing continuing education programs on gender and sexual diversity for health care providers. Successful efforts may be expected to improve trust and health care engagement among LGBTQ+ patients.

Future qualitative research should prioritize the inclusion of underrepresented subgroups, including bisexual, transgender, and non-binary individuals, and expand to under-researched cultural settings in Asia, the Middle East, Africa, and elsewhere. There is also a need to develop research frameworks and tools grounded in the MSM and intersectionality theory to enhance cross-study comparability and theoretical rigor.

Policymakers and health care authorities should make concerted efforts to advance LGBTQ+-inclusive health policies and national care standards. Moreover, these policies and standards should be integrated into medical and nursing curricula and accreditation and evaluation frameworks to systematically reduce barriers to care and promote health equity for LGBTQ+ populations.

### Conclusions

In this review, the health care experiences of LGBTQ+ adults were synthesized, with the results revealing persistent challenges such as intersecting stigma, heteronormative assumptions, structural barriers, and health care mistrust. These issues, which significantly limit access and equity, are ameliorated by culturally sensitive and affirming care environments. The findings emphasize that LGBTQ+ health care engagement is shaped by systemic, interpersonal, and sociocultural forces. To improve outcomes, health care systems must move beyond individual-level interventions and implement structural reforms that recognize and support diverse identities.

### Artificial Intelligence Usage

During the data synthesis process, ChatGPT-4.0 (OpenAI) was used as a language-based tool to assist with the preliminary organization and naming of thematic categories. All of the substantive synthesis, interpretation, and final naming decisions were made exclusively by the human research team to ensure accuracy, relevance, and reflexivity.

## Supplementary Material
