# Supplementary material for: Health Care Experiences Among LGBTQ+ Adults: A Qualitative Systematic Review and Meta-Synthesis
Source: J Nurs Res. 2026 May 19;34(3):e463. doi: 10.1097/jnr.0000000000000750 (PMC13200891; doi:10.1097/jnr.0000000000000750)
Supplement: Supplementary file 1 [file jnr-34-e463-s001.docx]

**Appendix**

*Summary of the Included Studies*

| First Author (Year)/Region | Data Collection | Data  Analysis | Phenomenon of Interest | Participants' Sexual Orientation | | Sample Size | Key Findings (Themes) |  |  |  |
| --- | --- | --- | --- | --- | --- | --- | --- | --- | --- | --- |
| Bradford (2016)/  United States | Focus groups | Thematic  analysis (TA) | Needs and transitions related to healthy aging | Lesbian | | 26 | Economic vulnerability; forced concealment of sexual orientation; fear of discrimination; limited access and affordability of long-term care; preference for LGBTQ-friendly and culturally competent providers. | | |  |
| Bristowe (2018)/  United Kingdom | Interviews | TA | End-of-life care experiences | Gay, lesbian, bisexual, transgender | | 40 | Individualized care needs of older adults; lack of LGBTQ-inclusive services; assessment of sexual orientation disclosure; intersectional discrimination; selective disclosure of care-relevant information to providers. | | | |
| Burton (2020)/  United States | Interviews | TA | Perspectives and experiences of older LGBT individuals toward healthcare providers | Gay, not disclosed | 10 | | Sexual orientation disclosure; religious-based prejudice and discrimination; attentiveness and support toward LGBTQ issues; secondary trauma; community-based aging care needs. | | | |
| Czimbalmos (2022)/ Finland | Interviews, Focus groups | TA | Healthcare challenges of migrant transgender individuals | Trans men, trans women, trans masculine, nonbinary | | 14 | Awareness of privilege or marginalization; lack of cultural competence in care; differences in socioeconomic conditions; intersectional discrimination across sexual orientation, migration status, social class, age, and race. | |  |  |
| Greene (2019)/ United States | Interviews, Focus groups | Content  analysis | Experiences and preferences for coming out to providers | Lesbian, bisexual, queer, other | | 33 | Providers' heteronormative assumptions; how and whether providers ask about sexual orientation; anticipated discrimination; decision-making around disclosure; LGBTQ-friendly clinical settings. | |  |  |
| Hascher (2024)/ United States | Interviews | TA | Perspectives of sexual minority men on their healthcare experiences | Gay | | 43 | Enhancing patient safety and autonomy reduces stigma and discrimination; facilitates continued treatment engagement and healthcare equity. | |  |  |
| Her (2016)/  United States | Interviews | Grounded  theory | Mental health service experiences of Hmong LGBT individuals | Gay, bisexual, lesbian | | 6 | Isolation at work and in social life; community and internalized stigma toward mental health; search for culturally competent therapists. | |  |  |
| Higgins (2019)/ United States | Interviews, Focus groups | TA | Facilitators and barriers to contraception among queer women | Queer women or non-heterosexual | | 33 | Exclusion of queer women from contraception outreach; discrimination, stigma, and sexual violence hinder access to care; coming out processes facilitate sexual empowerment. | |  |  |
| Holland (2021)/ United States | Interviews | TA | Campus and women’s center counseling experiences of sexual assault survivors | Gay, lesbian, bisexual, transgender | | 40 | Cisgender men and gender-diverse individuals face barriers accessing women’s centers; concern over inclusivity; therapists perceived as lacking skills in supporting LGBTQ and sexual assault survivors. | |  |  |
| Hoskin (2016)/ United States & Canada | Open-ended survey responses | TA | Concerns about sexual health screening and care | Trans women, trans men, queer, others | | 926 | Discomfort with forced disclosure and fear of provider reactions; disrespect from heteronormative providers; those with low gender identity clarity less likely to seek sexual health screening; desire for providers sharing gender identity. | |  |  |
| Hoyt (2020)/ United States | Focus group | Content  analysis | Healthcare experiences related to prostate cancer | Gay men | | 11 | Heterosexism and stigmatization from providers; empathy, trust, and openness foster positive patient experiences, while poor communication and a "salesman-like" demeanor result in negative experiences; intersectional discrimination based on sexual orientation, illness status, and adverse life experiences. | |  |  |
| Joudeh (2021)/ United States | Interviews | TA | Experiences accessing healthcare services in rural areas | Gay, lesbian, bisexual, transgender | | unknown | Intersectional discrimination and healthcare access barriers related to geographic location (U.S. South), religion, and race among sexual and gender minorities. | |  |  |
| Kelsall-Knight (2020)/ United Kingdo | Interviews | Narrative  research | Healthcare experiences of non-biological lesbian mothers seeking care for their children | Lesbian | | 3 | Feeling marginalized in healthcare encounters; affirming sexual orientation as identity protection; questioning provider professionalism; application of family constellation therapy. | |  |  |
| Kielhold (2024)/ United States | Interviews | TA | Role of healthcare providers in the care of Black sexual minority men living with HIV | Gay and bisexual men | | 27 | Supportive relationships with providers foster continuity of care and affirm patients’ self-worth, while poor communication weakens trust and willingness to seek care. | |  |  |
| Kulshreshtha (2020)/ India | Focus group | Interpretative phenomenolo-gical analysis | Mental health needs and healthcare experiences of sexual minorities | Gay, lesbian, bisexual, pansexual, asexual, transgender | | 8 | Negative consequences of disclosing sexual orientation; mental health and social challenges among queer individuals; stigmatization by mental health professionals; embodied experiences of self and identity; sociocultural and ecological influences; individual resilience trajectories. | |  |  |
|  |  |  |  |  |  |  |  |  |  |  |
|  |  |  |  |  |  |  |  |  |  |  |
| Legere (2016)/ Canada | Interview | Content  analysis | Medical experiences and healthcare barriers among reproductive cancer patients | Lesbian, bisexual women | | 6 | The influence of meaningful social support on care experiences; the friendliness and inclusivity of cancer care providers; considerations around disclosing sexual orientation; unfriendly healthcare environments marked by heterosexism, biphobia, and heteronormativity. | |  |  |
| Logie (2018)/ Eswatini | Interview | TA | Healthcare barriers experienced by individuals living with HIV | Gay, bisexual, transgender individuals | | 51 | Processes of social exclusion and marginalization; intersecting discrimination based on law, healthcare, HIV, and sexual minority identity; empowerment and community support in HIV care. | |  |  |
| Müller (2017)/ South Africa | Interviews; Focus groups | TA | Opportunities for HIV counseling, testing, and treatment among LGBT individuals | Gay, lesbian, queer, transgender individuals | | 16 | Healthcare resource gaps: (1) Availability: lack of public health services for general and LGBT-specific needs; (2) Accessibility: providers refusing to treat LGBT patients; (3) Acceptability: moral judgment and dissatisfaction with LGBT identities, pressure to participate in religious practices; (4) Quality of care: lack of understanding of LGBT identities and health needs; (5) Discrimination by providers and fear of retaliatory harm. | |  |  |
| Nadarzynski (2017)/ United Kingdom | Interview; Focus groups | Framework analysis | Perceptions of HPV and HPV vaccination among men who have sex with men (MSM) | Gay men | | 32 | Targeted HPV vaccination among MSM perceived as discriminatory and unjust, contributing to bias and marginalization. Lack of sexual orientation disclosure and limited access to sexual health clinics were identified as barriers. While most participants recognized benefits of the vaccine, overall vaccine acceptability remained low. Insufficient knowledge and perceived low need were major obstacles to successful implementation of HPV vaccination programs. | |  |  |
| Paine (2018)/ United States | Interviews | Retrospective analysis | Healthcare disparities among non-cisgender individuals | Non-binary | | 34 | Non-cisgender sexual minorities face more discrimination and healthcare avoidance than cisgender individuals. Providers often cite lack of knowledge on transgender care as a reason to deny treatment. Participants reported concealing their gender and sexual identity. | |  |  |
| Rana (2022)/ Canada | Focus groups | TA | Perceptions of STI testing services among gay, bisexual, and other men who have sex with men (GBM) | Cisgender men | | 27 | Key concepts included the need for more clinic locations and extended hours, use of online lab requisitions to reduce testing barriers, and the importance of professional, unbiased, compassionate, and competent care from providers. | |  |  |
| Smart (2022)/ United States | Interviews | Grounded  theory | Social determinants of health, healthcare experiences, and health priorities among transgender women of color | Transgender women | | 15 | Identified 20 themes across three domains: (1) Social determinants (family rejection, discrimination and violence, isolation, policy barriers, mistrust in healthcare, job insecurity, sex work, cost of care, transportation barriers, religious rejection, substance use); (2) Healthcare experiences (emotional burden during encounters, misgendering, provider discomfort or indifference, risk assumptions, use of non-medical or exploitative providers); (3) Health priorities (understanding healthcare, respect across services, inclusive gender-affirming care, integrated resources). | |  |  |
| Soinio (2020)/  Finland | Interviews | Content analysis | Healthcare experiences and expectations of lesbian and bisexual women | Lesbian, bisexual women | | 22 | Heteronormative assumptions by institutions and providers; lack of cultural competence; importance of LGBT-friendly and inclusive environments; considerations around sexual orientation disclosure; past discrimination affecting healthcare experiences. | |  |  |

*Note.* LGBTQ = lesbian, gay, bisexual, transgender, and queer; LGBT = lesbian, gay, bisexual, and transgender; HPV = Human Papillomavirus; MSM = Minority Stress Model; STI = sexually transmitted infections; GBM = gay and bisexual men.
